# Development and pilot testing of the 2019 Canadian Abortion Provider Survey

**DOI:** 10.1186/s40814-023-01279-1

**Published:** 2023-03-23

**Authors:** Regina M. Renner, Madeleine Ennis, Mahan Maazi, Sheila Dunn, Wendy V. Norman, Janusz Kaczorowski, Edith Guilbert

**Affiliations:** 1 Department of Obstetrics and Gynaecology, University of British Columbia, Suite 930, 1125 Howe Street, Vancouver, BC Canada V6Z 2K8 Canada; 2 Contraception and Abortion Research Team, Women’s Health Research Institute, BC Women’s Hospital and Health Centre, 4500 Oak Street, Vancouver, BC Canada V6H 3N1 Canada; 3 Department of Family and Community Medicine, University of Toronto, 27 King’s College Cir, Toronto, ON Canada M5S 1A1 Canada; 4 Department of Family Practice, University of British Columbia, 3Rd Floor David Strangway Building, 5950 University Boulevard, Vancouver, BC Canada V6T 1Z3 Canada; 5 Faculty of Public Health and Policy, London School of Hygiene & Tropical Medicine, 15-17 Tavistock Place, London, WC1H 9SH UK; 6 Department of Family and Emergency Medicine, Université de Montréal, Montreal, Pavillon Roger-Gaudry, 2900 Edouard Montpetit Blvd, Montreal, QC H3T 1J4 Canada; 7 Department of Obstetrics, Gynecology and Reproduction, Laval University, 2325 Rue de L’Université, Québec City, QC Canada G1V 0A6 Canada

**Keywords:** Surveys and questionnaires, Abortion, Induced, Health workforce, Pilot studies, Internet, Delphi approach

## Abstract

**Background:**

Substantial changes in abortion care regulations, available medications and national clinical practice guidelines have occurred since a 2012 national Canadian Abortion Provider Survey (CAPS). We developed and piloted the CAPS 2019 survey instrument to explore changes of the abortion provider workforce, their clinical care as well as experiences with stigma and harassment.

**Methods:**

We undertook development and piloting in three phases: (1) development of the preliminary survey sections and questions based on the 2012 survey instrument, (2) content validation and feasibility of including certain content aspects via a modified Delphi Method with panels of clinical and research experts, and (3) pilot testing of the draft survey for face validity and clarity of language; assessing usability of the web-based Research Electronic Data Capture platform including the feasibility of complex skip pattern functionality. We performed content analysis of phase 2 results and used a general inductive approach to identify necessary survey modifications.

**Results:**

In phase 1, we generated a survey draft that reflected the changes in Canadian abortion care regulations and guidelines and included questions for clinicians and administrators providing first and second trimester surgical and medical abortion. In phase 2, we held 6 expert panel meetings of 5–8 participants each representing clinicians, administrators and researchers to provide feedback on the initial survey draft. Due to the complexity of certain identified aspects, such as interdisciplinary collaboration and interprovincial care delivery differences, we revised the survey sections through an iterative process of meetings and revisions until we reached consensus on constructs and questions to include versus exclude for not being feasible. In phase 3, we made minor revisions based on pilot testing of the bilingual, web-based survey among additional experts chosen to be widely representative of the study population. Demonstrating its feasibility, we included complex branching and skip pattern logic so each respondent only viewed applicable questions based on their prior responses.

**Conclusions:**

We developed and piloted the CAPS 2019 survey instrument suitable to explore characteristics of a complex multidisciplinary workforce, their care and experience with stigma on a national level, and that can be adapted to other countries.

**Supplementary Information:**

The online version contains supplementary material available at 10.1186/s40814-023-01279-1.

## Background

In Canada, individuals have the right to safe, legal, and voluntary abortion care. Each year, over 80,000 surgical and medical abortions (MA) are provided in Canada [1]. In 2012, the first national Canadian Abortion Provider Survey (CAPS) captured responses from 78 abortion facility administrators and 178 physicians. Important findings included that there were relatively few abortion providers in Canada, most responding facilities and clinicians were non-hospital-based and focused the majority of their clinical care on abortion provision, rural urban abortion access disparities existed, and that MA contributed to less than 4% of all abortions [2, 3]. However, since 2012, substantial changes in the abortion care landscape have occurred; Health Canada approved mifepristone, the international gold standard drug for MA, and it became available in 2017 [4, 5]. In the same year, restrictive regulations around prescribing and dispensing mifepristone were removed [6] and subsequently the cost of the medication was covered in most provinces [7]. Cost of abortion care otherwise was already covered under Canada’s universal health care. Regulations now allow nurse practitioners (NPs) to independently provide first trimester MA care [8]. The Society of Obstetricians and Gynaecologists of Canada (SOGC) published updated evidence-based clinical practice guidelines for both medical and surgical abortions [9, 10]. To fill a knowledge gap, we aimed to develop the CAPS 2019 to assess the impact of these changes.

Based on our team’s clinical experiences and supported by emerging evidence [7], we hypothesized that the workforce has increased and that care has shifted away from primarily occurring in urban locations and abortion specific clinics to enhanced rural access and in primary care settings [7]. The aims of the CAPS 2019 study were to survey the multidisciplinary workforce and document the change in their characteristics and distribution compared to CAPS 2012; to assess the care they provide, i.e., characteristics of actual abortion practices as compared to the revised Canadian clinical practice guidelines, in both MA and surgical abortion practices; and to determine the extent abortion providers experienced harassment and stigma in their work, and explore their related resilience and retention. Our objective for this study was to develop the CAPS 2019 survey instrument to meet our aims (phase 1), validate the instrument using expert panels (phase 2), and pilot test the usability of the survey in a web-based format (phase 3).

## Methods

The CAPS 2019 survey was developed and conducted by members of the Contraception and Abortion Research Team–Groupe de recherche sur l’avortement et la contraception (CART-GRAC), a national, interdisciplinary, cross-sectoral research group that supports the provision of health services and implementation of policies to promote equitable access to high-quality family planning knowledge, methods, and health care services throughout Canada [11]. In this study, we aimed to develop a national cross-sectional survey, to explore changes in the abortion workforce, their clinical care and experience with stigma and harassment.

Chronologically, the survey development involved (1) development of the preliminary survey sections and questions, (2) content validation and feasibility of including certain content aspects via a modified Delphi Method with panels of clinical and research experts, and (3) pilot testing of the draft survey for face validity and clarity of language; assessing usability of the web-based Research Electronic Data Capture platform including the feasibility of complex skip pattern functionality [12]. Ethics approval for this study was obtained from the University of British Columbia’s Children’s and Women’s Hospital Research Ethics Board (H18-03,313).

### Phase 1: development of the preliminary survey sections and questions

We used the CAPS 2012 instrument as the basis for the 2019 survey. We leveraged the expertise of our multisectoral CART-GRAC members (including clinicians, workforce specialists, and researchers) in virtual working-group meetings to decide which survey changes would be required to capture changes in workforce and clinical practice. We refined the study population of interest to include non-physician clinicians and all types and aspects of clinical abortion care (first and second trimester surgical and first, second, and third trimester MA). In collaboration with our Institutional Research Board (IRB), the decision was made to anonymize the survey in order to maximize protection of participants’ personal identity. This was important due to the sensitive nature of abortion care.

We identified relevant publications to inform the required changes such as professional regulations for NPs, Health Canada regulations on mifepristone prescribing and dispensing [13, 14], mifepristone product monograph [5, 15], and the SOGC clinical practice guidelines [9, 10]. We mapped these publications to relevant survey sections and individual questions. The survey section on first trimester MA required major revisions as the mifepristone medication regimen only became available in Canada in 2017 and, therefore, was not included in the 2012 survey. We reviewed the USA first trimester MA section of the 2012 survey for questions we could use or adapt for our survey [16]. Due to the complexity of provincial variations in abortion related policies and service delivery between provider types (physicians, NPs, midwives, and administrators) some of the survey topics and questions required an iterative process of meetings and revisions until consensus was reached. The participants of the working-groups ensured we created relevant and concise questions, placed in appropriate sections of the survey and in a logical order to ensure understanding.

Additionally, we collaborated with the authors of the validated stigma and harassment scale we had used in the 2012 survey to update our survey section on stigma and harassment which included questions beyond the original scale [17, 18] exploring resilience and retention of providers.

Further considerations of the initial survey development included moving from a combined paper- and web-based survey sent to known abortion facilities to a web-based survey accessible via a generic survey link in order to reach a wider and largely unknown study population. This decision would also allow us to use branching logic that enabled respondents to only read questions that applied to them based on their prior answers, to minimize survey fatigue. Our instrument was available through the REDCap platform [12], a secure web-based meta-data-driven web application, housed at our partner research institute, BC Children’s Hospital Research Institute.

### Phase 2: content validation via a modified Delphi method with a panel of clinical experts

#### Panel assembly

We virtually convened clinical and research expert panels from across Canada, consisting of representatives of the study population, to examine face validity of the first version of the survey instrument, using a modified Delphi method (described below) [19–21]. Experts were primarily co-investigators or knowledge users of our study team, and were asked to invite colleagues to participate through a snowball sampling approach if we thought we needed further expert representation [22]. We held panels for each type of abortion care and each provider type and ensured representation of rural and urban, as well as hospital versus non-hospital based, specialists and generalist clinicians in order to optimize the survey. Depending on their clinical expertise, some experts attended multiple panel meetings.

#### The Delphi method

We used a Delphi method [19, 21] to consult a representative group of Canadian abortion providers to discuss and validate the initial survey questions and sections. We modified each of the rounds of Delphi expert input by revising the survey instrument after each of the panel sessions to reflect the input provided by that group before meeting with the next panel. Some of the survey sections (e.g., demographics) were reviewed by multiple panels, some only applied to one of the panels (e.g., second trimester surgical abortion). As some of the experts sat on multiple panels, they reviewed subsequent versions of some sections again as suggested in the Delphi method [20]. A final version of the survey instrument was created after all expert panel input was incorporated.

Each panel consisted of 5–8 clinicians and researchers who met through videoconference. At least two research team members (RR, WVN, SD, EG) moderated each meeting. The moderator, following a standard semi-structured interview guide, provided a project description followed by survey instrument information highlighting the changes compared to the 2012 survey instrument and subsequently guided the content validation process (see Table 1 for content validation and Supplement `1 for complete interview guide). Expert panelists reviewed each question for clarity and relevance. We invited them to give feedback on wording of questions and response options including their rationale, suggest non-essential questions to be removed, and provide feedback on whether the proposed questions and response options would feasibly capture the abortion related regulations and care delivery specific to their provider type (physician versus NP versus midwives versus administrator) and province. We reserved time for open-ended discussion, panelists to share their perceptions and experiences with care provision and implementation of mifepristone. The meetings were audio-recorded and transcribed.

**Table 1:** CAPS 2019 development panelist instructions for question analysis

General instructions
We would like you to review the questions with a focus on the following changes to the previous survey:
**1**. We plan to have an electronic survey only, which means that we can use branching logic and filter questions. This will limit the questions to the fewest and most appropriates per participant
**2**. In the 2012 survey, we focused on facilities with administrators as a unit as well as on providers. In the 2019 survey, we aim to capture the change in workforce which we predict includes many more solo practitioners. Therefore, we will use individual providers as the unit of measurement, rather than facilities
Assess individual items in the survey:
1. How did you find the wording of the question?
2. What are your thoughts on the purpose of the question? Elaborate if necessary: “purpose” as in “what is the question trying to ask?”
3. What are your thoughts on the correlation between the question and the responses listed for that question?
4. Were there any answers that you would have liked to have had as potential responses but which were not listed with the question? If so, what were these answers?
5. Were there any answers listed that you feel were irrelevant or unnecessary? If so, which were those answers?
General feedback for the survey:
1. What were the strengths of the survey? What were the weaknesses of the survey?
2. Was the survey presented in a logical manner? If not, what would be a more logical progression for the survey?
3. Were there any missing topics or questions that you feel might be beneficial for our study? Please elaborate

*CAPS* Canadian Abortion Provider Survey

#### Response analysis

Our panel meeting facilitators (RR, SD, WVN, EG) independently reviewed transcripts to identify emerging themes. Together they conducted a content analysis of the transcripts in a recursive fashion to identify common and conflicting viewpoints in a stepwise analysis. We examined stratified groups (Physician’s versus NPs versus midwives versus administrators and groups from different provinces) for similarities and differences. This entire analysis was conducted to refine the scope of the abortion workforce and service delivery we would feasibly be able to capture in this survey, to remove unneeded questions and modify existing ones, and to identify important topics missed in the first version of the survey.

Our usability analysis involved an iterative process to change the format of survey questions. For example, we had asked panelists to provide feedback on preferences between free text answers versus check boxes with number intervals or Likert scales. We ensured standardization of language between survey sections, consistent use of language, and consistency between Likert scale directions throughout the survey, i.e., from strongly disagree to strongly agree or from less to more often. Discussions further aimed at clarifying ambiguous terms or concepts and the possible consequence of different answers from respondents; i.e., regarding service delivery models, such as being the “most responsible provider” versus working in a “shared care model”, or working in a clinic versus office versus facility.

In the next step of our analysis, we used a general inductive approach to identify needed content modifications to questions and answer options. For example, in the context of regulations not allowing independent midwifery abortion care, the midwifery service delivery models across the country proved to be too heterogeneous to be feasibly captured in a survey rather than in an interview. We therefore removed questions related to abortion practice by midwives from our survey. In the context of the evolving COVID-19 pandemic during the subsequent piloting phase, co-investigators emphasized the importance to clarify that our survey asked about 2019 and pre-COVID care, but also to add a question at the end of the survey exploring how the pandemic affected respondents’ abortion care.

#### Finalizing the bilingual survey instrument and creating a web-based survey

Following the response analysis, the core co-investigator working group who had developed the initial survey draft reviewed a revised version of the survey instrument and iteratively suggested further modifications until we reached consensus. Thereafter, we professionally translated the survey into French, and the Francophone investigators on our team (MSW and EG) reviewed the French survey for correct translation of medical terminology and relevance for Francophone respondents.

The bilingual survey instrument was entered into REDCap. We programmed questions that determined the branching logic or were considered essential as mandatory and respondents could not continue until they had answered that question, while other questions could be skipped by the respondents if they chose. Detailed programming in collaboration with the REDCap technical team was further needed so that the electronic survey allowed respondents to choose answer options in the intended fashion and to avoid mistakes; i.e., radio buttons if only one answer was asked for versus an option to check all answer options that applied to a respondent. We further programmed “check all that apply”, and answer options that were incompatible with other answer options to the same question as an “action tag” which automatically unselected the other answer options to that question. When applicable, we displayed multiple sub-questions and answer options to a combined question stem in a table format called “matrix”. We used the options of soft and hard coding of number answers to decrease the chance of non-sensical answers to questions involving percentages or numbers such as gestational age of a pregnancy. “Soft-coding” allowed respondents to override a pop-up window with a warning that the answer was outside of the expected range (for example usual upper gestational age limit for a clinical care aspect), while “hard coding” did not allow entering the number at all (for example percentage greater than 100). Generally, we aimed to format the survey in a web user-friendly design, e.g., minimize the need to scroll on a screen, and allowed respondents to create a return code if they wanted to complete the survey in multiple sittings.

To protect respondent confidentiality as requested by our IRB, the main study section did not include personal identifiers. However, in a separate survey section that was not linked to the data, respondents could provide their email address for remuneration purposes ($50 gift certificate) or to be contacted with study results or future research.

Web-based surveys increase the risk of fraudulent responses, especially when anonymous and recruiting via a generic link, which pose a threat to their data integrity [23, 24]. Offering financial incentives as well as conducting research on socially or politically sensitive topics are additional risk factors for fraud [25]. Therefore, we took measures to decrease the risk for fraud with careful choices of our study design [23, 24]. To help prevent fraud from robots we added a CAPTCHA to the beginning of the survey. In our initial survey question, we asked respondents to confirm that they had not taken the survey before. We planned to monitor incoming responses for possible fraud. Based on the literature, this consisted of confirming sensical combinations between a few select questions in the demographics [26]. We describe our detailed fraud detection strategy in a separate manuscript [27].

### Phase 3: pilot testing of the draft survey

We conducted extensive piloting in order to assess usability, face validity, and clarity of language throughout the online survey as well as usability of advanced REDCap functionality to display questions and response options, and feasibility of complex survey components such as skip pattern functionality. First, we distributed the link for the web-based draft survey among co-investigators and study team members, each of whom represented one or more aspects of the target study population. This included individuals who were a part of phase 2, as well as individuals who had not yet seen the survey instrument. Along with the survey link we provided instructions to respond with specific recommendations or issues that arose. In an iterative approach, as we received suggestions, a team of 6 study investigators and research assistants (RR, MSW, MG, MM, MV, and ME) regularly met via videoconference to reach consensus on how to revise the online survey. Thereafter, the same team systematically piloted all REDCap functionality on multiple internet browsers and corrected errors. This cycle of testing, revising, and retesting the survey instrument in REDCap continued until we identified no further concerns.

## Results

### Phase 1: development of the preliminary survey sections and questions

Between April and August 2019 we held 4 multisectoral (including approximately 20 clinicians, workforce specialists and researchers) co-investigators (many of which were clinician experts in abortion care themselves) and knowledge user meetings to iteratively refine the survey population, inform the initial survey draft and agree to the changes compared to the CAPS 2012. Table 2 highlights the differences between the surveys’ study populations, types of abortion care and survey design. In 2012, the majority of abortion services were provided in facilities that focused on abortion care. We primarily surveyed administrators of these facilities in CAPS 2012, and asked them to pass on the survey to at most 5 clinicians in their facility. Due to the availability of mifepristone for first trimester MA since 2017, we anticipated a move of abortion care from facilities with a focus on abortion care into primary care clinics. In order to capture this change, we considered the clinician as the primary unit of recruitment rather than the clinic administrator and expanded the survey sections for individual providers while shortening the clinic administrator section. Data on second and third trimester MA which are provided in hospitals was scarce in our 2012 survey. The 2012 survey section on second trimester MA had not included many details and due to our recruitment focus on non-hospital-based facilities in 2012, we did not capture this type of abortion care well. To include this care more comprehensively in 2019, we expanded the second trimester MA section, added specific third trimester language and placed the questions in a clinician rather than administrator section. We further modified our recruitment strategy to include individual clinicians in both hospitals and non-hospital based settings.

**Table 2:** CAPS sections, topics and design in 2012 compared to 2019

Year	2012	2019
Study population	i. Abortion care facility administrators (who received survey invitation) ii. Up to five physicians per facility	i. Individual abortion providers/clinician (physicians including maternal fetal medicine subspecialists, NPs) ii. Clinic administrators
Types of abortion care	i. First trimester MA with *methotrexate* ii. First trimester surgical abortion iii. Second trimester surgical abortion iv. Second trimester MA	i. First trimester MA with *mifepristone* ii. First trimester surgical abortion iii. Second trimester surgical abortion iv. Second trimester MA v. Third trimester MA
Survey design	i. Primarily paper–pencil with separate booklets for administrators versus physicians ii. Web-based available with one section for administrators and one for physicians	Web-based with multiple sections and using branching logic to allow respondent to only see questions that applied to them based on their prior answers

*CAPS* Canadian Abortion Provider Survey, *MA* medical abortion

The first version of the survey instrument consisted of 6 sections and up to 58 questions per sub-section (however, due to the branching logic respondents would not see all questions). The survey sections were demographics (Sect. 1), clinical abortion practices (Sect. 2; divided into subsections of first trimester MA, first trimester surgical abortion, second trimester surgical abortion, and second/third trimester MA), administrator (Sect. 3), diverse populations (Sect. 4), stigma and resilience (Sect. 5), and remuneration and future research (Sect. 6). We used questions from our CAPS 2012 survey when possible; however, the majority required modifications due to the changes in the workforce, new regulations, availability of mifepristone for MA and updated guidelines.

### Phase 2: content validation via a modified Delphi method with a panel of clinical experts

In September and October 2019, we held 7 expert panel meetings, composed of 5–8 participants each. These represented abortion providers who were: physicians, NPs, midwives, and administrators; from both urban and rural communities across Canada in addition to researchers. Table 3 depicts a list of meetings, their topics, panelist characteristics and key findings of the content analysis including the survey components found to be not feasible. Recurring themes between panels were (1) the desire to capture as much information as possible, including constructs such as cultural or structural competence [28, 29], while avoiding survey fatigue; (2) balancing pros and cons of open-ended questions as a tool to explore respondent’s experiences and attitudes that however might increase survey fatigue and would also require qualitative data analysis; (3) offering questions and answer options that were applicable to variations in service delivery between provinces, rural and urban respondents as well as different provider types; and (4) reaching consensus on clarity of language around terminology and their definitions.

**Table 3:** Expert panel meetings in preparation of the CAPS 2019

Meeting	Topic	Panelist characteristics	Key findings of content analysis
1	First trimester MA	*n* = 7 FP, Ob&Gyn, RN; administrator; rural and urban; private office and abortion clinic based	• Definition of most responsible provider as the person who signs the prescription in order to avoid double reporting of procedures • Definition of clinical scenarios/terminology such as pregnancy of unknown location to ensure respondents correctly understand question
2	First trimester surgical abortion	*n* = 6 FP, Ob&Gyn; administrator; rural and urban; academic and community hospital based	• Clarification of variations in service delivery around for example variations in ultrasound and counseling provision in order to provide sensical answer options • Definition of prophylactic antibiotic use versus as clinically indicated to ensure respondents correctly understand answer options
3	Registered midwives (RM)	*n* = 7 RM, Ob&Gyn, researcher	• RMs cannot independently prescribe mifepristone for first trimester MA • Regulations and practice around collaborative care between physicians and RMs are complex and highly variable. We will not adequately capture those in a survey and, therefore, RMs are not eligible to participate
4	Nurse practitioners (NP)	*n* = 8 RN, NP, Ob&Gyn, researcher	• Only NPs can independently prescribe mifepristone for first trimester MA. Therefore, they are eligible to participate in the survey • RNs cannot independently prescribe mifepristone. Regulations and practice around collaborative care between physicians or NPs and RNs are complex and highly variable. We will not adequately capture those in a survey and therefore RNs are not eligible to participate
5	Second trimester surgical abortion	*n* = 5 Ob&Gyn; administrator; rural and urban; academic and community hospital based	• Definition of abortion for life fetus rather than management of spontaneous intrauterine fetal demise in order to ensure respondents correctly identify their eligibility to that survey section and report accurate procedure numbers • Ensure consistent definitions and wording of question and answers between survey sections • Avoid redundancies between survey sections such as testing and management of Rhesus status
6	Stigma and harassment	*n* = 7 Ob&Gyn, SW, sociologist, researchers who developed validated stigma scale	• Replace the validated stigma scale we used in our 2012 survey with the updated validated scale of the same research team[17] • Adjust terminology from abortion providers to health care providers who offer abortion care to better fit how our study population might self-identify • Considerations to explore additional constructs of cultural [29] or structural competence [30] but decided that the first was not comprehensive enough and the latter not validated enough to be a good fit for this survey. Rather added a survey sections to explore respondents training about and approach to providing care for diverse populations
7	Second/third trimester MA	*n* = 6 general Ob&Gyn, MFM; administrator; rural and urban; academic and community hospital based	• Terminology of labour induction versus second trimester MA to avoid confusion among respondents • Definition of number of procedure as number of deliveries in order to avoid double counting MAs, as many labor inductions will involve multiple providers • Expanding answer options around consultation services to capture variations in service delivery between provider specialties, provinces, and rural versus urban hospitals • Meaningful Likert scale to answer questions around frequency of use or strength of recommendations around clinical care aspects

*CAPS* Canadian Abortion Provider Survey, *MA* medical abortion, *Ob&Gyn* obstetrician and gynecologist, *FP* family physician, *RN* registered nurse, *NP* nurse practitioner, *RM* registered midwife, *SW* social work, *MFM* maternal–fetal-medicine sub-specialist

### Phase 3: pilot testing of the draft survey

In January 2020, we piloted the web-based survey with approximately 25 co-investigators and knowledge users, all of whom were clinicians and within the target population. Based on their feedback we completed minor modifications, including some wording changes for further clarity, correcting spelling errors and changing the order of questions in February 2020. From March to June 2020 we piloted and optimized REDCap programming and found that advanced program functionality made it feasible to achieve a user friendly survey display and experience including complex skip pattern logic. The CAPS 2019 was originally set to launch in the spring 2020, as information and experiences related to 2019 abortion care would be readily recalled. However, due to the COVID-19 pandemic, our team recognized that clinicians would not have the time and energy to respond to a survey. Therefore, the survey launch date was postponed to July 2020. The final survey instrument of our cross-sectional, national, bilingual, web-based CAPS 2019 including the anticipated time it would take to complete the individual sections is summarized in Table 4.

**Table 4:** Final CAPS 2019 survey instrument sections and time to complete them

Section	Content	Description	Time (minutes) to complete the section
1	Demographics	Anonymized workforce characteristics including age, gender, profession, clinical specialty, geographic location of clinical practice, type of abortion care provided, and percentage of clinical work focusing on contraception and abortion	15
2	Clinical abortion practices: ◦ First trimester MA ◦ First trimester surgical abortion ◦ Second trimester surgical abortion ◦ Second/third trimester MA	Pre-abortion testing, procedure techniques and medications, post-abortion follow-up; number of abortions, clinic characteristics	15 15 15 15
3	Administrators	Anonymized characteristics of facility, its workforce and clinical care, number of abortions	10
4	Diverse populations	Adaptation of care for diverse patient populations, such as cultural/ethnic origins, gender/identity	5
5	Stigma and resilience	Experience with stigma and harassment, and their associated resilience	5
6	Remuneration and future research	Respondents could provide email address to receive remuneration, study results or future research information	1
Respondents only see the questions that apply to the demographics or services they indicated in prior questions. All respondents are asked to complete Sects. 1, 4, 5, and 6. Clinicians will be asked to complete Sect. 2. The greater the range of abortion care a respondent provides (covered in Sect. 2), the longer it will take to complete the survey. Administrators will be asked to complete Sect. 3 instead of Sect. 2			

*CAPS* Canadian Abortion Provider Survey, *MA* medical abortion

## Discussion

We developed a cross-sectional, national, bilingual, web-based survey instrument that we piloted and revised in a rigorous and iterative approach until face validity and usability were assured. The CAPS 2019 explored the abortion provider workforce, the care they provide and their experiences with stigma and harassment. It was developed in order to capture changes compared to 2012 and after the implementation of mifepristone for first trimester MA in Canada in 2017 as well as other regulatory changes and updated clinical practice guidelines [5, 9, 10, 14, 15]. We navigated the challenge that, while we aimed to build on the 2012 survey to preserve question validity and to be able to directly compare our results, the survey instrument needed considerable adaptation and inclusion of additional content to reflect the Canadian context and changes in abortion care since 2012.

Our study process resulted in a more comprehensive survey than either our CAPS 2012 [2, 3], or prior U.S. iterations [16, 30, 31] from which the CAPS 2012 had been adapted. We moved the primary unit of recruitment and most survey questions from the abortion facility administrator to the individual clinician. We included NPs providing first trimester MA and expanded the questions on second and third trimester MA. As in the CAPS 2012, we relied on a validated scale to explore respondents’ experience with stigma and harassment [17]. Our national and multisectoral team allowed us to recognize that some topics, such as interdisciplinary collaborative care between midwives or nurses and physicians were too heterogeneous and complex to be feasibly captured by a survey, but rather required other research methods such as interviews, which could be conducted in a separate project.

Limitations: Overall our option to use previously fielded or validated questions was limited. The anticipated shift from abortion facility-based services to primary care, which differs from the service delivery in the USA [16]. Limited the degree to which we could use the previously fielded U.S. survey mifepristone first trimester MA questions. Similarly, the shift from administrator to clinician questions required modification of prior questions. Phase 3 pilot testing focused on the usability and face validity of the online survey instrument. We chose not to include specific measurements such as Cronbach alpha coefficients to assess internal consistency, test–retest reliability testing, or factor analysis to describe variability among items. While the co-investigators participating to pilot test our survey were clinical experts in the field and within the target population, it is possible that their feedback was influenced by their role on the study team, and that they were not naïve to the survey. We think, however, that our extensive panel group meetings and piloting in addition to using previously [32] fielded or validated scales when possible provided good reassurance in regard to question validity. Additionally, we previously successfully developed and piloted several effective survey instruments using a multiphase approach usually forgoing quantitative testing of specific question parameters [32–35].

Strengths of our study included the expansion of the study population to include previously understudied aspects of abortion care such as second and third trimester MA. Further, the web-based functionality allowed us to use branching logic to mitigate the length of our survey and to limit survey fatigue. REDCap additionally offered the opportunity to program individual answer options or combinations of answers in a way that limited respondent errors.

## Conclusions

The CAPS 2019 development study used a rigorous multiphase approach to develop a comprehensive and nationally representative survey instrument of the abortion workforce, their clinical care and experience with stigma and harassment. It may provide the foundation for surveys in other countries. Results from our survey will provide data to inform knowledge translation activities of decision-makers in Canada aiming to improve equitable access to high quality abortion care.

## Supplementary Information

**Additional file 1: Supplement 1.** Interview guide for focus groups.

## Data Availability

The datasets generated and analyzed during the current study are available from the corresponding author upon reasonable request. The final version of the Canadian Abortion Provider Survey is available from the corresponding author upon reasonable request.

## Abbreviations

CAPS: Canadian Abortion Provider Survey
MA: Medical abortion
Ob&Gyn: Obstetrician and gynecologist
FP: Family physician
RN: Registered nurse
NP: Nurse practitioner
RM: Registered midwife
SW: Social work
MFM: Maternal-fetal-medicine sub-specialist
SOGC: Society of Obstetricians and Gynaecologists of Canada
IRB: Institutional Research Board

## References

[1] Canadian Institute for Health Information. Induced abortions reported in Canada 2018. In: CIHI, editor. Ottawa, Canada: Government of Canada; 2020. Available at [https://www.cihi.ca/en/access-data-reports/results?query=abortion&Search+Submit=] Accessed 25 Jan 2020.

[2] Norman WV, Guilbert ER, Okpaleke C (2016). Abortion health services in Canada: results of a 2012 national survey. Can Fam Physician.

[3] Guilbert ER, Hayden AS, Jones HE (2016). First-trimester medical abortion practices in Canada: National survey. Can Fam Physician.

[4] Dunn S, Cook R (2014). Medical abortion in Canada: behind the times. CMAJ..

[5] Health Canada. Regulatory decision summary: MIFEGYMISO. In: Canada H, editor. Ottawa, Canada: Government of Canada; 2015. Copy on CART-GRAC website of the original government web page, now archived and not available.

[6] Munro S, Guilbert E, Wagner M-S (2020). Perspectives among Canadian physicians on factors influencing implementation of mifepristone medical abortion: a national qualitative study. Ann Fam Med.

[7] CART-GRAC (Contraception & Abortion Research Team-Groupe de recherche sur l’avortemont et la contraception). Canadian Abortion Providers Support-Communauté de pratique canadienne sur l’avortement (CAPS-CPCA) Community of Practice. Established 2017 in Vancouver, British Columbia, Canada: University of British Columbia; 2017.

[8] College of Nurses of Ontario. What NPs should know about Mifegymiso. Available at: http://www.cno.org/en/news/2017/july-2017/what-nps-should-know-about-mifegymiso/. Accessed 24 Mar 2019; 18 Jul 2017.

[9] Costescu D, Guilbert E (2018). No. 360-induced abortion: surgical abortion and second trimester medical methods. J Obstet Gynaecol Can..

[10] Costescu D, Guilbert E, Bernardin J (2016). Medical Abortion. J Obstet Gynaecol Can.

[11] Contraception & Abortion Research Team--Groupe de recherche sur l’avortemont et la contraception (CART-GRAC) (2019). CART-GRAC.

[12] BC Children’s Hospital Research. BCCHR REDCap datasystem. BCCHR; 2019 [cited 2019 Nov 11]. Available at https://rc.bcchr.ca/.

[13] Blaze Baum K. Health Canada eases restrictions on abortion pill Mifegymiso. The Globe & Mail. Phillip Crawley; 2017. Available at: https://www.theglobeandmail.com/news/national/health-canada-eases-restrictions-on-abortion-pill-mifegymiso/article36860275/ Accessed 28 Sept 2021.

[14] Health Canada. Mifegymiso. Health Canada updates prescribing and dispensing information for Mifegymiso. 7 Nov 2017. In: Canada Go, editor. Ottawa: Government of Canada; 2017.

[15] Health Canada (2016). Mifegymiso Product monograph including patient medication information.

[16] Jones HE, O’Connell White K, Norman WV, Guilbert E, Lichtenberg ES, Paul M (2017). First trimester medication abortion practice in the United States and Canada. PLoS ONE.

[17] Martin LA, Debbink M, Hassinger J, Youatt E, Eagen-Torkko M, Harris LH (2014). Measuring stigma among abortion providers: assessing the abortion provider stigma survey instrument. Women Health.

[18] Harris LH, Martin L, Debbink M, Hassinger J (2013). Physicians, abortion provision and the legitimacy paradox. Contraception.

[19] Hsu C-C, Sandford BA (2007). The Delphi technique: making sense of consensus. Pract Assess Res Eval.

[20] Iqbal S, Pipon-Young L. The Delphi method. The psychologist; 2009 [cited 2019 Aug 27]. Available from: https://thepsychologist.bps.org.uk/volume-22/edition-7/delphi-method.

[21] Murry JW, Hammons JO (1995). Delphi: a versatile methodology for conducting qualitative research. Rev High Educ.

[22] Johnson TP (2014). Snowball Sampling: Introduction.

[23] Ballard AM, Cardwell T, Young AM (2019). Fraud detection protocol for web-based research among men who have sex with men: development and descriptive evaluation. JMIR Public Health Surveill.

[24] Teitcher JE, Bockting WO, Bauermeister JA, Hoefer CJ, Miner MH, Klitzman RL (2015). Detecting, preventing, and responding to "fraudsters" in internet research: ethics and tradeoffs. J Law Med Ethics.

[25] Quach S, Pereira JA, Russell ML (2013). The good, bad, and ugly of online recruitment of parents for health-related focus groups: lessons learned. J Med Internet Res.

[26] Kramer J, Rubin A, Coster W (2014). Strategies to address participant misrepresentation for eligibility in Web-based research. Int J Methods Psychiatr Res.

[27] Ennis M, Contadriopoulos D, Albert A (2021). Development of a Fraud Detection Algorithm for the Canadian Abortion Provider Survey.

[28] Schim SM, Doorenbos AZ, Miller J, Benkert R (2003). Development of a cultural competence assessment instrument. J Nurs Meas.

[29] Metzl JM, Petty J (2017). Integrating and assessing structural competency in an innovative prehealth curriculum at Vanderbilt University. Acad Med.

[30] White KO, Jones HE, Lavelanet A (2019). First-trimester aspiration abortion practices: a survey of United States abortion providers. Contraception.

[31] White KO, Jones HE, Shorter J (2018). Second-trimester surgical abortion practices in the United States. Contraception.

[32] Devane C, Renner RM, Munro S (2019). Implementation of mifepristone medical abortion in Canada: pilot and feasibility testing of a survey to assess facilitators and barriers. Pilot Feasibility Stud.

[33] Wong E, Leslie JJ, Soon JA, Norman WV (2016). Measuring interprofessional competencies and attitudes among health professional students creating family planning virtual patient cases. BMC Med Educ.

[34] Jia L, Norman WV (2021). Contraception practices among women on opioid agonist therapy. J Obstet Gynaecol Can.

[35] Wong M, Soon J, Zed P, Norman W (2014). Development of a survey to assess the acceptability of an innovative contraception practice among rural pharmacists. Pharmacy.

