# Supplementary material for: Development and pilot testing of the 2019 Canadian Abortion Provider Survey
Source: Pilot Feasibility Stud. 2023 Mar 23;9:49. doi: 10.1186/s40814-023-01279-1 (PMC10034882; doi:10.1186/s40814-023-01279-1)
Supplement: Supplementary file 1 — Additional file 1: Supplement 1. Interview guide for focus groups. [file 40814_2023_1279_MOESM1_ESM.docx]

**Supplement 1** Interview guide for focus groups

Project Title: Canadian Abortion Providers Survey 2019 – Survey Pilot

Firstly, I would like to **thank you** for your participation in today’s working group for First Trimester Medical Abortion Providers.

**PROJECT DESCRIPTION:**

The Canadian Abortion Provider Survey (CAPS) is a CIHR funded, 4 year, national study that will examine the following aims:

**Aim 1:** To document the change in characteristics and distribution of the abortion care workforce since the 2012 Canadian Abortion Provider Survey;

**Aim 2:** To assess the quality of care, i.e., characteristics of actual abortion practices as compared to the revised Canadian clinical practice guidelines, in both medical abortion (MA) and surgical abortion (SA) practices and

**Aim 3:** To determine to what extent providers experience harassment and stigma in their work and explore their related resilience and retention.

**INSTRUMENT INFORMATION:**

The survey has the following sections:.

Section 1 addresses questions about demographics of abortion providers;

Section 2 addresses questions about clinical abortion practices;

Section 3 addresses questions about experiences as a provider of abortion services;

Section 4 addresses administrative support related to abortion care;

**CONTENT VALIDATION PROCESS:**

This working group session will take up to 90 minutes. We are looking for your feedback on whether the instrument will be able to capture what it means to measure, as well as on the clarity of language and usability of the questions themselves and optional answers (importance, relevance and clarity). This will help us improve our survey for distribution to all physicians and allied health care professionals such as nurse practitioners and registered midwives currently providing medical abortion.

In order for us to remember and learn from your comments today, we will be recording your comments. Please be assured that we will treat our conversation and the recording as private and confidential.

We would like you to review the questions with the lens of the following changes to the previous survey:

1. We plan to have an electronic survey only, which means that we can use branching logic and filter questions. This will limit the questions to the fewest and most appropriates per participant.
2. In the 2012 survey we focused on facilities with administrators as a unit as well as on providers. In the 2019 survey we aim to capture the change in workforce which we predict includes many more solo practitioners as the unit of measurement, rather than larger facilities.

**Assess individual items in the survey:**

1. How did you find the wording of the question?
2. What are your thoughts on the purpose of the question? Elaborate if necessary: “purpose” as in “what is the question trying to ask?”
3. What are your thoughts on the correlation between the question and the options listed for that question?
4. Were there any options that you would like to have responded but not listed in the question? If so, what were these options?
5. Were there any options that you feel were unnecessary? If so, what were these options?

[After all items from the questionnaire have been completed, the participants will engage in a general feedback section, comprised of the following questions.]

**General feedback for the survey:**

1. What were the strengths of the survey? What were the weaknesses of the survey?
2. Was the survey presented in a logical manner? If not, what would be a more logical progression for the survey?
3. Were there any missing topics or questions that you feel may be beneficial for our study? Please elaborate.
4. If you were requested to complete this survey in the community as a physician, nurse practitioner or registered midwife are there any barriers that would prevent you from completing the survey?
5. Other comments?

**Closing comments**

Once again, thank you for your participation in today’s expert panel working group. Your valuable insight will be very useful as we continue to refine our survey instruments.
